# Pivotal role of inter-organ aspartate metabolism for treatment of mitochondrial aspartate-glutamate carrier 2 (citrin) deficiency, based on the mouse model

**DOI:** 10.1038/s41598-019-39627-y

**Published:** 2019-03-12

**Authors:** Takeyori Saheki, Mitsuaki Moriyama, Eishi Kuroda, Aki Funahashi, Izumi Yasuda, Yoshiko Setogawa, Qinghua Gao, Miharu Ushikai, Sumie Furuie, Ken-ichi Yamamura, Katsura Takano, Yoichi Nakamura, Kazuhiro Eto, Takashi Kadowaki, David S. Sinasac, Tatsuhiko Furukawa, Masahisa Horiuchi, Yen How Tai

**Affiliations:** 1 Department of Hygiene and Health Promotion Medicine, Kagoshima University Graduate School of Medical and Dental Sciences, Kagoshima, Kagoshima, Japan; 2 Laboratory for Yamamura Projects, Institute for Resource Development and Analysis, Kumamoto, Kumamoto, Japan; 3 Laboratory of Integrative Physiology in Veterinary Sciences, Osaka Prefecture University, Izumisano, Osaka Japan; 4 Department of Internal Medicine, Teikyo University, Tokyo, Japan; 5 Department of Diabetes and Metabolic Diseases, Graduate School of Medicine, The University of Tokyo, Tokyo, Japan; 6 Alberta Children’s Hospital Research Institute, Department of Medical Genetics, Cumming School of Medicine, University of Calgary, Calgary, Alberta Canada; 7 Department of Molecular Oncology, Kagoshima University Graduate School of Medical and Dental Sciences, Kagoshima, Kagoshima, Japan; 8 Citrin Foundation, Singapore, Singapore

## Abstract

Previous studies using citrin/mitochondrial glycerol-3-phosphate (G3P) dehydrogenase (mGPD) double-knockout mice have demonstrated that increased dietary protein reduces the extent of carbohydrate-induced hyperammonemia observed in these mice. This study aimed to further elucidate the mechanisms of this effect. Specific amino acids were initially found to decrease hepatic G3P, or increase aspartate or citrulline levels, in mGPD-knockout mice administered ethanol. Unexpectedly, oral glycine increased ammonia in addition to lowering G3P and increasing citrulline. Subsequently, simultaneous glycine-plus-sucrose (Gly + Suc) administration led to a more severe hyperammonemic state in double-KO mice compared to sucrose alone. Oral arginine, ornithine, aspartate, alanine, glutamate and medium-chain triglycerides all lowered blood ammonia following Gly + Suc administration, with combinations of ornithine-plus-aspartate (Orn + Asp) or ornithine-plus-alanine (Orn + Ala) suppressing levels similar to wild-type. Liver perfusion and portal vein-arterial amino acid differences suggest that oral aspartate, similar to alanine, likely activated ureagenesis from ammonia and lowered the cytosolic NADH/NAD^+^ ratio through conversion to alanine in the small intestine. In conclusion, Gly + Suc administration induces a more severe hyperammonemic state in double-KO mice that Orn + Asp or Orn + Ala both effectively suppress. Aspartate-to-alanine conversion in the small intestine allows for effective oral administration of either, demonstrating a pivotal role of inter-organ aspartate metabolism for the treatment of citrin deficiency.

## Introduction

Aspartate (Asp)-glutamate (Glu) carrier 2 (AGC2) (a.k.a., Citrin) deficiency (CD)^1^, caused by mutations in *SLC25A13*^2^ encoding citrin, the liver-type AGC2^3^, has been established as the cause of adult-onset type II citrullinemia (CTLN2), neonatal intrahepatic cholestasis (NICCD) in patients during their first year of life, and of other presentations including failure to thrive and dyslipidemia (FTTDCD), pancreatitis, hepatoma and non-alcoholic steatohepatitis. The variable symptomatology is believed to arise from that fact that citrin, functioning as an AGC, plays important roles not only in supplying Asp from mitochondria to the cytosol for urea, protein and nucleotide syntheses, but also in transporting cytosolic NADH reducing equivalents into mitochondria as a member of malate-Asp shuttle. The latter role of citrin is related to the most characteristic finding in CD patients, their unique dietary predilection^4,5^; the average carbohydrate intake in Japanese CD patients is demonstrably lower than that of age-, sex- and ethnic-matched controls^4^. This has been hypothesized to be due to an underlying carbohydrate toxicity in CD from transiently-accumulated cytosolic NADH. It is known that standard treatment of hyperammonemia using a low protein/high carbohydrate diet leads to further exasperated hyperammonemia in CD patients^6^, as does infusions of high concentration glucose and glycerol-plus-fructose as a treatment for brain edema, which have led to consciousness disturbances and even death in some cases^1,7^. An established CD mouse model, the citrin (Ctrn)/mitochondrial glycerol-3-phosphate dehydrogenase (mGPD) double-knockout (KO)^8^, also shows hyperammonemia under fed conditions, which is exacerbated by oral sucrose (Suc) administration. As a result of inter-species differences in hepatic glycerophosphate shuttle activity between humans and mice, the requirement of the second mGPD-KO in combination with the Ctrn-KO allowed for the creation of a more phenotypically suitable CD mouse model compared to the single Ctrn-KO mouse.

Use of a low protein/high carbohydrate synthetic diet has also led to a marked decrease in food intake in our established CD mouse model, resulting in a loss of body weight that was ameliorated by increasing the protein content of the diet^9^. The same effect of increased protein was achieved by substituting single amino acids or other substances into the mouse chow, including amino acids such as alanine (Ala) and Glu, as well as sodium pyruvate (Pyr) or medium-chain triglycerides (MCT)^9^. The concomitant increase in hepatic glycerol 3-phosphate (G3P), a marker indicative of an increased cytosolic NADH/NAD^+^ ratio^10^, was also suppressed by such supplementation^9^. The effectiveness of one of these supplements on ureagenesis, sodium Pyr, was further proven by additional liver perfusion experiments^11^.

Although the effect of amino acids previously tested appeared to be non-specific, since both Ala and sodium Glu were similarly effective in our previous studies^9^, we decided to systematically test the effects of a larger subset of amino acid supplements in the present study, with the goal of identifying the mechanism or mechanisms of action for specific amino acids on important parameters in CD.

## Results

### Effects of supplementation on hepatic G3P, Asp and citrulline in mGPD-KO mice administered 5% ethanol

**Figure 1:**
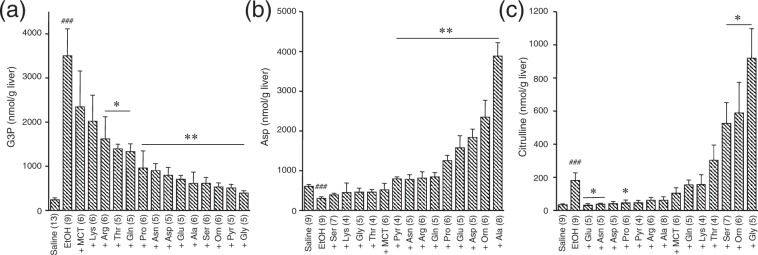
Effects of amino acid supplementation on hepatic glycerol 3-phosphate (G3P; panel (a)), aspartate (Asp; panel (b)), and citrulline (**c**) levels in mGPD-KO mice administered 5% ethanol. Amino acid solutions (1 M; with the exception of glutamine and asparagine at 0.5 M) was enterally administered with 5% ethanol (20 ml/kg bw), and the liver was removed by freeze-cramp procedure 1 h after administration. Data are expressed as mean ± SEM. Asterisks (*P < 0.05, and **P < 0.01) denote statistical differences compared to the levels following administered ethanol alone; ^###^P < 0.001, statistical difference between saline and ethanol administered groups.

To first look for effects of supplementation of amino acids (and other substances) on the hepatic G3P level (i.e., to look for effects that may be specific to an altered NADH/NAD^+^ ratio), as well as on their ability to alter urea-cycle intermediate levels independent of a loss of citrin function (as would be present in the Ctrn/mGPD double KO mice), we tested various amino acids as well as Pyr and MCT for their ability to suppress the increased G3P observed in mGPD-KO mice enterally administered 5% ethanol by volume (all solutions were administered at a dose of 20 ml/kg body weight (bw)), as well as on increasing hepatic Asp and citrulline (Cit) levels. We chose to use mGPD-KO mice as a starting point as we had found previously that hepatic G3P was similarly increased by administration of ethanol or glycerol in both mGPD-KO as well as double-KO mice^12^. The hepatic G3P level was increased more than 10-fold above that of saline 1 hr after the administration of 5% ethanol in the mGPD-KO mice (Fig. 1(a)), but that the simultaneous administration of 1 M solutions (20 mmol/kg bw) of glycine (Gly), ornithine (Orn), serine (Ser), Ala, Glu, Asp, asparagine* (Asn; *0.5 M was used at a dose of 10 mmol/kg bw) or proline (Pro) as well as Pyr all significantly suppressed the elevated G3P level. Lesser effects were observed for glutamine (Gln)* (*0.5 M was used), threonine and arginine (Arg), while no significant effect was observed for lysine or 5% MCT (1 g/kg bw). These results indicate that the effect of specific amino acids, similar to the effect of sodium Pyr, was unlikely to be affecting glucose or sugar metabolism directly, but seemed to act on NADH metabolism.

The results of this study also showed which supplements may have a potential benefit for increasing hepatic Asp (Fig. 1(b)) and decreasing hepatic Cit (Fig. 1(c)), since cytosolic Asp is deficient and Cit accumulates in CD due to the loss of citrin as the AGC2. The hepatic Asp level was significantly decreased by administration of 5% ethanol in the mGPD-KO mice but increased by simultaneous administration of either Ala, Orn, Asp, Glu, Pro, Gln, Arg, Asn or Pyr. The levels even exceeded those of the control (i.e., saline-treated mGPD-KO mice) level in the cases of Ala, Orn, Asp, Glu or Pro (Fig. 1(b)).

Hepatic Cit was also significantly increased by administration of 5% ethanol in the mGPD-KO mice. The increased Cit was further augmented by simultaneous administration of Gly, Orn or Ser, and in contrast, significantly decreased by administration of Glu, Asn or Pro (Fig. 1(c)). Unlike the simultaneous administration of Orn, however, that is expected to directly increase urea cycle function (and decrease ammonia levels), the increased hepatic Cit level following enteral administration of Gly or Ser was associated with increased ammonia production (Supplemental Fig. S1).

### Effect of simultaneous administration of Gly with Suc or ethanol on blood ammonia, and identification of a new induced hyperammonemic state

**Figure 2:**
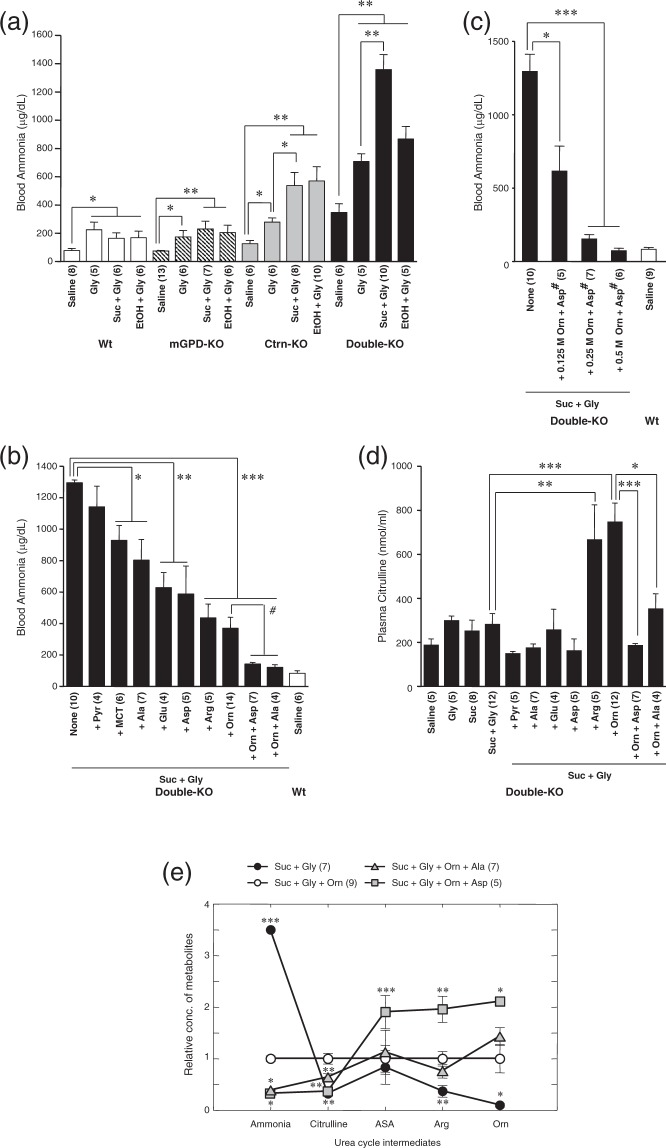
Effects of oral supplementation indicated on blood ammonia (panels (a), (b) and (c)) and plasma citrulline (d), and cross-over point analysis of urea cycle intermediates (e). (**a**) Effects of 1 M Gly administered with or without 20% sucrose (Suc) or 5% ethanol (EtOH) (20 ml/kg bw) on blood ammonia in all four mouse genotypes: wild type (wt; white bar), mGPD-KO (hatched bar), Ctrn-KO (gray bar) and Ctrn/mGPD double-KO (black bar). (**b**) Effects of 0.5 M amino acids (Ala, Glu, Asp, Arg or Orn; 10 mmol/kg bw), 0.5 M Pyr (10 mmol/kg bw), 5% MCT (1 g/kg bw) or combination of amino acids (each 10 mmol/kg bw) on blood ammonia levels increased by administration of 20% Suc + 1 M Gly (total 20 ml/kg bw) in double-KO mice, with wt mice administered saline as a reference level. (**c**) Dose response of Orn plus Asp (Orn + Asp^#^; L-aspartate salt of L-ornithine was used) on blood ammonia. The concentrations of Orn + Asp^#^ were 0.125 M (2.5 mmol/kg bw), 0.25 M (5 mmol/kg bw) or 0.5 M (10 mmol/kg bw), and administered with 20% Suc + 1 M Gly. (**d**) Effects of administration of amino acids and other substances indicated in the figure on plasma citrulline in Ctrn/mGPD double-KO mice. (**e**) Each of hepatic or blood metabolite concentrations under Suc + Gly + Orn was set at 1 and the metabolite concentrations under the other conditions were calculated and plotted. The concentration of blood ammonia was used as Ammonia in the figure. Data are expressed as mean ± SEM. Asterisks (*P < 0.05, **P < 0.01, and ***P < 0.001) denote statistical differences between indicated groups by an unpaired t-test (panels (a), (b), (c) and (d)). Asterisks (*P < 0.05, **P < 0.01, and ***P < 0.001) denote statistical differences from the baseline of Suc + Gly + Orn.

We next tested the effect of Gly in combination with 20% Suc (4 g/kg bw) or 5% ethanol in all four genotypes: wild-type (wt), mGPD-KO, Ctrn-KO and Ctrn/mGPD double-KO mice (Fig. 2(a)). The administration of Gly increased blood ammonia levels in all the genotypes tested, while blood ammonia levels were further increased by simultaneous administration of Suc or ethanol only in Ctrn-KO and double-KO mice. Furthermore, the blood ammonia level exceeded 1000 μg/dl following the administration of 20% Suc plus 1 M Gly in only the double-KO mice. In relative terms, the effect of simultaneous Gly + Suc administration was both much greater and more consistent than that produced previously in the double-KO mice by administration of Suc alone^8,10^, suggesting that this newly discovered inducible-hyperammonemic state could serve as an improved hyperammonemic model to search for effective supplements beneficial in CD.

### Ameliorating effects of Orn, Asp and Ala, with crossover point analysis

To further identify specific amino acids that may be most effective in altering the hyperammonemia in CTLN2 patients, we tested the candidate amino acids that were found to be most effective at both decreasing hepatic G3P and increasing hepatic Asp content within the context of this new induced hyperammonemic state (Fig. 1(a,b)): namely Ala, Glu, Orn and Asp. We excluded any further investigation of Ser even though it also showed a positive effect in decreasing hepatic G3P, since it increased hepatic Cit (Fig. 1(c)) as well as blood ammonia (Supplementary Fig. S1) similar to Gly, as shown above. In addition, we added Pyr, MCT and Arg as candidate supplements as well, since those have been found to be effective previously in mouse experiments^8,10^ or clinically^13–17^.

Figure 2(b) shows the effects of all of the substances tested on blood ammonia levels increased by administration of Suc + Gly in double-KO mice. Pyr failed to decrease blood ammonia while MCT showed a barely significant decrease (P = 0.048) in blood ammonia levels. All of the other amino acids tested (Ala, Glu, Asp, Arg and Orn) showed clear and significant effects of decreasing the blood ammonia level in double-KO mice induced by the administration of Suc + Gly. Since Arg and Orn are both urea cycle intermediates, but Arg showed a more modest effect in decreasing hepatic G3P and increasing hepatic Asp when compared to Orn, we used Orn as the first candidate to test combinations with other amino acids, namely Orn + Asp or Orn + Ala. Both combinations of amino acids further decreased the blood ammonia level to essentially wt saline-treated levels (Fig. 2(b)). Figure 2(c) shows a clear dose-response effect of the combination of Orn + Asp (it should be noted that in this experiment, salt-free forms of Orn and Asp were used, which do not contain molar equivalents of hydrochloride or sodium, respectively; shown as Orn + Asp^#^) on blood ammonia levels in the double-KO mice.

Plasma Cit levels were also a useful marker for identifying differences in the site of action for Orn, Asp and Ala, as shown in Fig. 2(d). Plasma Cit was increased by administration of either Arg or Orn separately, but the level was largely decreased by the further addition of Asp or Ala to Orn, indicating that the point of action of the latter two amino acids was distinct from Orn and Arg in that Asp and Ala may be specifically supplying cytosolic Asp in the liver. This was further proven by crossover point analysis of the hepatic urea cycle intermediates as shown in Fig. 2(e): The relative baseline formed by Suc + Gly + Orn was crossed-over at the steps between blood ammonia and Cit in the case of Suc + Gly, most likely at the step of ornithine carbamoyltransferase (OCT), and at the step of argininosuccinate synthetase (ASS) between Cit and argininosuccinate (ASA) by Suc + Gly + Orn + Asp, indicating that for those steps, OCT and ASS were activated by Orn and Asp, respectively. Lastly, it is worth noting that Ala appeared to have an additional effect between Arg and Orn.

### Effects of Ala and Asp on ureagenesis from ammonium chloride, and changes in lactate-to-pyruvate ratio in perfused liver

**Figure 3:**
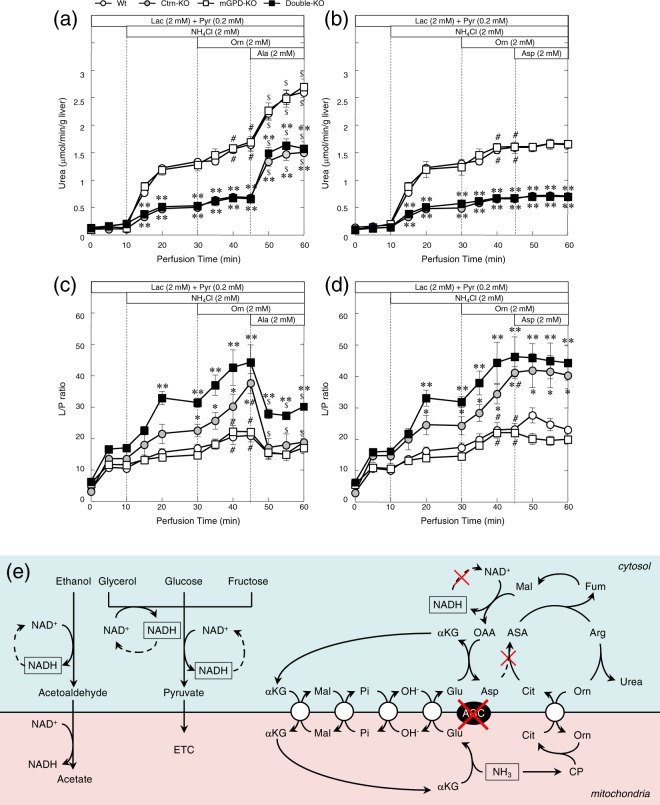
Effect of Ala (panels (a) and (c)) and Asp (panels (b) and (d)) on ureagenesis (panels (a) and (b)), and changes in lactate-to-pyruvate (L/P ratio; panels (c) and (d)) in perfused liver from wt (white circle), mGPD-KO (white square), Ctrn-KO (gray circle) and Ctrn/mGPD double-KO (black square) mice. (**e**) Schematic diagram of ureagenesis pathway from ammonia without citrin (AGC2) in relation to mitochondria and sugar and ethanol metabolism. The results are expressed as means ± SEM for 5-6 independent experiments. *P < 0.05, and **P < 0.01 denote statistical differences from wt mice. ^#^P < 0.05 and ^$^P < 0.05 denote statistical differences from the level at perfusion time 30 and 45 within the same genotypes, respectively. Abbreviations used are, αKG, α-ketoglutarate; ASA, argininosuccinate; Cit, citrulline; CP, carbamoylphosphate; ETC, electron transport chain; Fum, fumarate; OAA, oxaloacetate; Mal, malate; Pi, inorganic phosphate.

Using the liver perfusion system to further confirm our observations of the effects of Ala and Asp, the rates of ureagenesis were activated by the addition of ammonium chloride in all mouse genotypes, although the levels were significantly lower in livers from Ctrn-KO and double-KO mice when compared with the livers from wt and mGPD-KO mice. Furthermore, addition of Orn further increased ureagenesis in the livers of wt and mGPD-KO mice, but had little effect in Ctrn-KO and Ctrn/mGPD double-KO mice (Fig. 3(a)). However, ureagenesis was enhanced by the addition of Ala in the liver of all mouse genotypes (Fig. 3(a)), while the addition of Asp had no effect on urea production (Fig. 3(b)).

Analysis of the perfusate lactate and Pyr levels clearly showed increases in the lactate-to-pyruvate (L/P ratio), the index of cytosolic NADH/NAD^+^ ratio, in the livers from Ctrn-KO and the double-KO mice in response to ammonium chloride, and the L/P ratio was further augmented by the addition of Orn in the livers of Ctrn-KO and double-KO mice (Fig. 3(c)). However, it was not changed in the livers from wt and mGPD-KO mice, and only marginally increased by the addition of Orn in those mice (Fig. 3(c)). The addition of Ala also suppressed the ratio within the livers from all mouse genotypes (Fig. 3(c)), while in contrast, almost no change in the L/P ratio was observed following the addition of Asp in the livers from any of the mouse genotypes (Fig. 3(d)). The analysis of the liver perfusate L/P ratio also supported our hypothesis about the relationship between ureagenesis under CD and the metabolic effect of sugars and other substances which produce cytosolic NADH (Fig. 3(e)). Ureagenesis from ammonia is still possible under CD if the NADH formed during recycling of oxaloacetate (OAA) is oxidized. Intake of sugar or ethanol, infusion of high concentration of glucose, or infusion of glycerol and fructose, all increase cytosolic NADH which inhibits regeneration of OAA and therefore cytosolic Asp, resulting in inhibition of ASS step and accumulation of ammonia.

### Asp metabolism in the small intestine detected with portal vein-arterial differences in plasma amino acid concentrations

**Figure 4:**
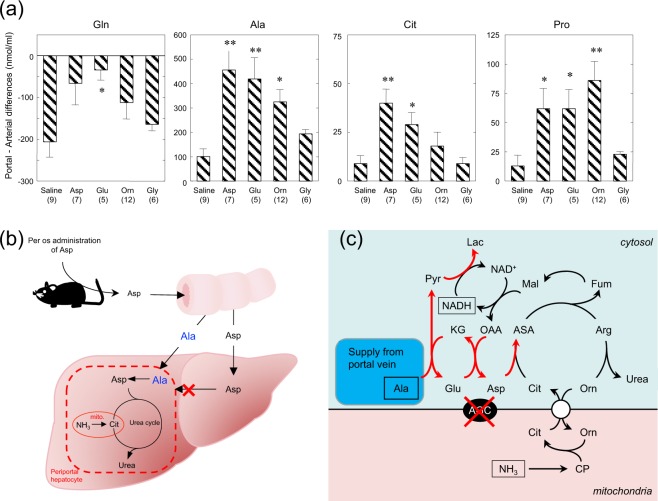
Portal vein-arterial differences in the plasma concentration of Gln, Ala, Cit and Pro 1 hr after administration of saline, Asp, Glu, Orn or Gly (**a**), schematic diagram of Asp metabolism after enteral administration within the small intestine and liver (**b**), and postulated metabolic pathway of Ala in periportal hepatocytes (**c**). Asp, Glu, Orn or Gly (20 ml/kg bw; 10 mmol/kg bw) were enterally administered to mGPD-KO mice, and 1 hr after the administration, blood was collected simultaneously from portal vein and abdominal aorta for the portal vein-arterial difference. Minus and plus values denote uptake by, and release from, the portal vein, respectively. Number of mice are shown in parentheses. *P < 0.05 and **P < 0.01 denote differences compared to saline.

The effectiveness of Asp as an orally-administered supplement was at first surprising to us, because of the conflicting results on ureagenesis in the perfused liver from Ctrn-KO mice^11^ and the report by Stoll *et al*.^18^ showing that Asp together with Glu and α–ketoglutarate are transported into perivenous hepatocytes but not periportal hepatocytes where urea cycle enzymes are specifically located. The reason why Asp was effective by oral administration and not liver perfusion, however, can be explained with results reported by Neame and Wiseman^19^, Parsons and Volman-Mitchell^20^ and Windmueller and Spaeth^21^ showing that enterally-administered Asp is converted mainly into Ala in the small intestine and then transported via the portal vein to the liver. In the present study, enteral administration of specific amino acids caused increases in each administered amino acid within the arterial circulation, and clear portal-arterial differences (Supplementary Fig. S2) demonstrating an uptake of Gln and output of Ala, Cit and Pro (Fig. 4(a)). Our additional findings clearly demonstrate significant increases in Ala output after enteral administration of Asp, Glu, and Orn, but not by Gly, as compared with saline (Fig. 4(a)); namely, enterally-administered Asp, Glu, and to a lesser extent Orn as well, are being converted to Ala in the small intestine, suggesting that the positive effect of Orn on enhancing ureagenesis appears to be more than simply increasing urea-cycle intermediate levels. The Ala formed and supplied to the periportal hepatocytes can then be reconverted back to Asp by coupling of the two cytosolic aminotransferases, and the formed Pyr can then oxidize malate to OAA, reinitiating ureagenesis under CD (Fig. 4(b,c)).

## Discussion

In this report, we have clearly shown that Ala, Glu and Asp were almost equally effective in ameliorating a newly-described induced hyperammonemia in our CD model, the Ctrn/mGPD double-KO mice. First, while evaluating the effect of oral administration of a variety of amino acids on decreasing hepatic G3P that was increased by simultaneous administration of ethanol in the mGPD-KO mice (Fig. 1(a)), we specifically showed that Gly had a dramatic effect on both increasing hepatic Cit (Fig. 1(c)) as well as increasing blood ammonia (Supplementary Fig. S1). Taking advantage of this newly discovered hyperammonemic effect of Gly, we established a new induced hyperammonemic model with highly elevated blood ammonia in the double-KO mice by administration of Gly with Suc (Fig. 2(a)).

The model was suitable to test the anti-hyperammonemic effects of other supplements including amino acids, MCT and sodium Pyr. Amino acids including Orn, Arg, Glu, Ala and Asp, and MCT demonstrated positive effects (Fig. 2(b)). Although singular supplements were not sufficient to completely prevent the induced hyperammonemic state, combinations of either Orn + Ala or Orn + Asp were equally effective in decreasing blood ammonia to control levels in the double-KO mice (Fig. 2(b)). The additional effects of Ala and Asp seemed reasonable, since both Ala and Asp increased hepatic Asp in the mGPD-KO mice (Fig. 1(b)), decreased the hepatic Cit that was increased by administration of Orn through activation of the OCT enzymatic step (Fig. 2(d,e)), and activated the ASS step as demonstrated by the crossover point analysis (Fig. 2(e)). However, since Asp was known to be taken up by perivenous hepatocyte but not periportal hepatocytes^18^ where urea cycle enzymes are located, and since we have actually shown that Asp was not effective to activate ureagenesis in the perfused liver of Ctrn-KO mice^11^, the results shown in Fig. 2(b–e) were discordant with these previous findings. We further showed that Asp was indeed ineffective in ureagenesis from ammonium chloride in the perfused liver of the double-KO mice as well (Fig. 3(b)); however, this apparent discrepancy was resolved by the experiments shown in Fig. 4(a). Namely, amino acid metabolism in the small intestine analyzed by simultaneous sampling of portal vein and arterial blood demonstrated release of Ala from the small intestine after administration of Asp, Glu and Orn (Fig. 4(a)), indicating that Asp was being converted to Ala in the small intestine where it could be supplied to periportal hepatocytes and activate ureagenesis (Figs 3(a), 4(b,c)). These results clearly show a pivotal role of the small intestine in inter-organ Asp metabolism for ureagenesis in CD. Similar to Asp, Glu could also participate in ureagenesis through metabolic conversion to Ala in the small intestine and supply Asp for ureagenesis within periportal hepatocytes.

It was an unexpected result that Orn also produced Ala in the small intestine, although Orn showed the most effect in increasing hepatic Asp in mGPD-KO mice (Fig. 1(b)); the increase in hepatic Asp from Orn is considered of mitochondrial origin due to the mitochondrial localization of ornithine aminotransferase, and not being transported to the cytosol under Ctrn-deficient conditions. The need for simultaneous administration of Asp or Ala, however, indicates insufficient supply of Asp from Orn for ureagenesis in the double-KO mice.

The previously demonstrated therapeutic effect of Pyr in CD^13–15^ is well accordant with the two recent studies on the effect of Pyr on cell proliferation under suppressed mitochondrial electron-transport chain function showing a significant role of Asp biosynthesis by the mitochondria^22,23^. In the present study, however, Pyr did not show any therapeutic effect on hyperammonemia induced by oral administration of Suc + Gly (Fig. 2(b)). This may be because the cytosolic NADH/NAD^+^ ratio under the administration of Suc + Gly was not substantially increased, and in fact, was likely somewhat suppressed by Gly as shown in Fig. 1(a); the hepatic G3P content was 1370 ± 520 nmol/g liver (n = 5; P < 0.05 vs saline) under Suc + Gly administration, as compared with 769 ± 301 nmol/g liver (n = 16) under saline and 3150 ± 1330 nmol/g liver (n = 8) under only Suc administration, respectively^10,14^. This also suggests that Gly was the major source of blood ammonia, as used by Marini *et al*.^24^, under which Pyr was not effective. The mechanism of hyperammonemia caused by Suc + Gly remains unresolved, although the Gly cleavage system may be activated by a decreased mitochondrial NAD(P)H/NAD(P)^+^ ratio^25^ caused by the combination of absent malate-Asp shuttle activity and inhibition of glycolysis leading to decreased Pyr intake into mitochondria, as evidenced by suppressed TCA cycle intermediates^10^. Furthermore, we note that administration of Gly to mGPD-KO mice decreased the plasma Gln and Glu levels (Supplementary Fig. S3), suggesting that Gln and Glu breakdown may be being activated, each possibly by ammonia^26^ derived from Gly and by a decreased ATP^27^ level under the conditions^14^ used, respectively. An alternative possibility for the high blood ammonia and lower hepatic Gln from Gly administration is that Gly may be inhibiting glutamine synthetase in the perivenous cells leading to reduced ammonia handling^28^. However, if the former theory^25^ is correct, a mechanism of action for MCT in reducing the blood ammonia level may be related to releasing the inhibition on the Gly cleavage by providing NADH and ATP.

## Conclusion

In conclusion, our data have revealed a pivotal role of inter-organ metabolism between the small intestine and liver of Asp and Glu in CD by supplying Asp via Ala to periportal hepatocytes, in addition to a detrimental effect of a large amount of Gly administered with Suc by increasing blood ammonia. Furthermore, use of Orn + Asp, Orn + Ala, or Ala, Asp or Glu alone are all compelling candidate therapeutic supplements for CTLN2, and potentially other clinical presentations caused by CD, warranting further clinical investigation in CD patients.

## Materials and Methods

### Animal care

All animals received humane care, and all the procedures were approved by the Ethical Committees for Animal Experimentation at Kagoshima University, Tokushima Bunri University, Osaka Prefecture University and Kumamoto University. All experiments were conducted in compliance with the ARRIVE guidelines.

### Animals

All wt, Ctrn-KO, mGPD-KO and Ctrn/mGPD double-KO mice used were congenic on the C57BL/6J background. Mice were generated using the breeding scheme described previously by Saheki *et al*.^8^. Briefly, mGPD-KO and double-KO mice were obtained by mating heterozygous Ctrn-KO/homozygous mGPD-KO (i.e., Ctrn^+/−^/mGPD^−/−^) mice, while wt and Ctrn-KO mice were generated by mating heterozygous Ctrn-KO (i.e., Ctrn^+/−^/mGPD^+/+^) mice. Genotyping was performed with DNA extracted from ear punches using procedures specific for each of the targeted mutations in the Ctrn-KO^29^ and mGPD-KO^30^ mice, as described in Saheki *et al*.^12^.

### Treatment

All mice were maintained at a constant temperature (23 ± 1 °C) on a 12-hour light/dark cycle (light on, 8:00 am to 8:00 pm) with free access to water and CE2 chow (24.9% protein, 4.6% fat, and 51% nitrogen-free extracts providing 343 kcal/100 g; CLEA Japan, Tokyo, Japan). Mice, both male and female, used for the experiments were analyzed between 60 and 160 days of age. In the experiments to examine the metabolic alterations in liver of the mice, fed mice were sacrificed between 10:00 am and 11:00 am by cervical dislocation one hour after enteral administration of a test solution (5 volume % ethanol, amino acids and other substances at the concentrations indicated) at a standard dose of 20 ml/kg bw by gastric tube. Basic and acidic amino acids, in addition to Pyr, were administered as hydrochloride or sodium salts, respectively, with the exception of the experiments described in Fig. 2(c), where the free forms of Orn and Asp, or Orn Asp salt, were used. Livers were quickly removed, freeze-clamped between aluminum tongues, pulverized under liquid nitrogen and homogenized in 3% perchloric acid. Following the centrifugation of the samples at 10,000×g for 10 min at 4 °C, the supernatants were neutralized with 1 M sodium bicarbonate and used for liquid chromatography or enzymatic analyses (as described below).

In the experiments to test blood ammonia and plasma amino acids, blood was taken from heart, or simultaneously from portal vein and abdominal aorta, under anesthesia with somnopentyl (60 mg/kg i.p.).

The rationale for using a 20 ml/kg gavage volume of 1 M solutions* of amino acids was based on the addition of protein, casein, to the AIN-93M diet (deemed sufficient for mature rodent maintenance by the American Institute for Nutrition (1994)) that resulted in increased food intake and body weight in the double-KO mice, as reported previously^9^. Added protein in the form of hydrolysed casein (i.e., tryptone) or single amino acid supplementation such as Ala or sodium Glu had similar effect, indicating that certain amino acids derived from digested protein might be responsible, if given in sufficiently large enough amounts. Therefore, all test supplements were administered simultaneously with Suc or other solutions at a standard dose of 20 ml/kg, maximizing the largest achievable dose. *In the case of Gln and Asn, whose solubility is less than one molar, we prepared solutions of 0.5 M.

### Liver perfusion experiments

Liver perfusion was performed on male mice essentially as described previously^11^. In each experiment, the liver was perfused for 30 min in the absence of exogenous substrates before various combinations of substrates were added (see figure legends). The perfusate, after passage through the liver, was collected at the indicated times. At the end of perfusion, the liver was freeze-clamped in liquid nitrogen and stored at −70 °C for hepatic amino acid, lactate and Pyr as well as urea analyses.

### Analytical procedures

The hepatic G3P concentrations were determined using an enzymatic method^31^. Hepatic amino acid concentrations were determined by liquid chromatography/mass spectrometry (LC/MS) (Acquity ultra performance liquid chromatography/Acquity TQD tandem-quadrupole mass spectrometer; Waters, Milford, MA, USA) after solid phase extraction and derivatization^32^ using the EZ:faast amino acid analysis kit (Phenomenex Ltd., Los Angeles, USA). Blood ammonia concentration was determined with Ammonia Test Wako (Wako Pure Chemicals, Osaka, Japan). In the perfusion experiments, concentrations of ammonia, urea, lactate and Pyr were quantified enzymatically^33–35^.

### Statistical analysis

All data are presented as means ± SEM. The difference between two group means was evaluated using the Student’s t-test.

### Materials

Chemicals used were: ammonium chloride, L-Arg hydrochloride, L-Ala, ethanol (99.5%), L-Gln, Gly, L-Orn hydrochloride, L-Ser, sodium L-Asp, sodium hydrogen L-Glu, sodium L-lactate (solution), sodium Pyr, Suc, and L-threonine from Wako Pure Chemical Industries, Ltd., Osaka, Japan, or Nacalai Tesque, Inc., Kyoto, Japan; L-Orn L-Asp (L-ornithine L-aspartate salt) from Tokyo Chemical Industry Co. Ltd., Tokyo; L-lysine hydrochloride and L-Pro from Sigma-Aldrich Inc., Japan, Osaka, Japan; MCT (78.9% powder) from Kissei Pharmaceutical Co. Ltd, Nagano, Japan. Glutamate dehydrogenase, glycerol-3-phosphate dehydrogenase and lactate dehydrogenase were purchased from Sigma-Aldrich Japan, Osaka, Japan.

## Supplementary information

supplementary information
