# Supplementary material for: Pivotal role of inter-organ aspartate metabolism for treatment of mitochondrial aspartate-glutamate carrier 2 (citrin) deficiency, based on the mouse model
Source: Sci Rep. 2019 Mar 12;9:4179. doi: 10.1038/s41598-019-39627-y (PMC6414645; doi:10.1038/s41598-019-39627-y)
Supplement: Supplementary file 1 — supplementary information [file 41598_2019_39627_MOESM1_ESM.pdf]

Supplementary Information

Pivotal role of inter-organ aspartate metabolism for treatment of mitochondrial aspartate-glutamate carrier 2 (citrin) deficiency, based on the mouse model

Takeyori Sahki[1,2*], Mitsuaki Moriyama[3], Eishi Kuroda[1], Aki Funahashi[1], Izumi Yasuda[1], Yoshiko Setogawa[1], Qinghua Gao[1], Miharu Ushikai[1], Sumie Furuie[2], Ken-ichi Yamamura[2], Katsura Takano[3], Yoichi Nakamura[3], Kazuhiro Eto[4], Takashi Kadowaki[5], David S. Sinasac[6], Tatsuhiko Furukawa[7], Masahisa Horiuchi[1], and Yen How Tai[8]

[1]Department of Hygiene and Health Promotion Medicine, Kagoshima University Graduate School of Medical and Dental Sciences, Kagoshima, Kagoshima, Japan; [2]Laboratory for Yamamura Projects, Institute for Resource Development and Analysis, Kumamoto, Kumamoto, Japan; [3]Laboratory of Integrative Physiology in Veterinary Sciences, Osaka Prefecture University, Izumisano, Osaka, Japan; [4]Department of Internal Medicine, Teikyo University, Tokyo, Japan; [5]Department of Diabetes and Metabolic Diseases, Graduate School of Medicine, The University of Tokyo, Tokyo, Japan; [6]Alberta Children’s Hospital Research Institute, Department of Medical Genetics, Cumming School of Medicine, University of Calgary, Calgary, Alberta, Canada; [7]Department of Molecular Oncology, Kagoshima University Graduate School of Medical and Dental Sciences, Kagoshima, Kagoshima, Japan; [8]Citrin Foundation, Singapore

***Corresponding author:** Takeyori Saheki, MD, PhD, Department of Hygiene and Health Promotion Medicine, Kagoshima University Graduate School of Medical and Dental Sciences, 8-35-1 Sakuragaoka, Kagoshima 890-8544 Japan.
Telephone number; +81-99-275-5291,
Fax: +81-99-265-8434
E-mail: takesah@gmail.com

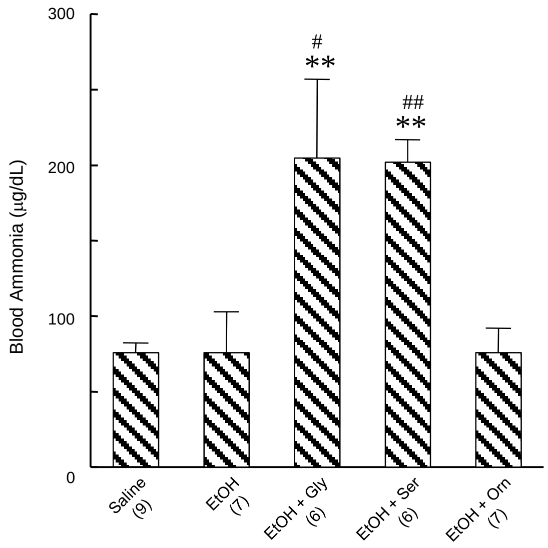

Supplementary Figure S1. Effect of administration of 5% ethanol (EtOH) and further addition of 1M glycine, serine or ornithine (20 ml/kg) on blood ammonia in mGPD-KO mice.

Experimental procedures are the same as described in Fig. 2(a). Data are presented as mean ± SEM. Asterisks (**P<0.01) and (#P<0.05 and ##P<0.01) denote statistical differences from the levels when administered saline and ethanol, respectively. Number of mice are shown in parenthesis.

Blood ammonia level was increased by enteral administration of 1M glycine plus 5% ethanol (EtOH) (20ml/kg bw) and 1M serine plus 5% ethanol, but not by 1M ornithine plus ethanol, or ethanol alone in mGPD-KO mice.

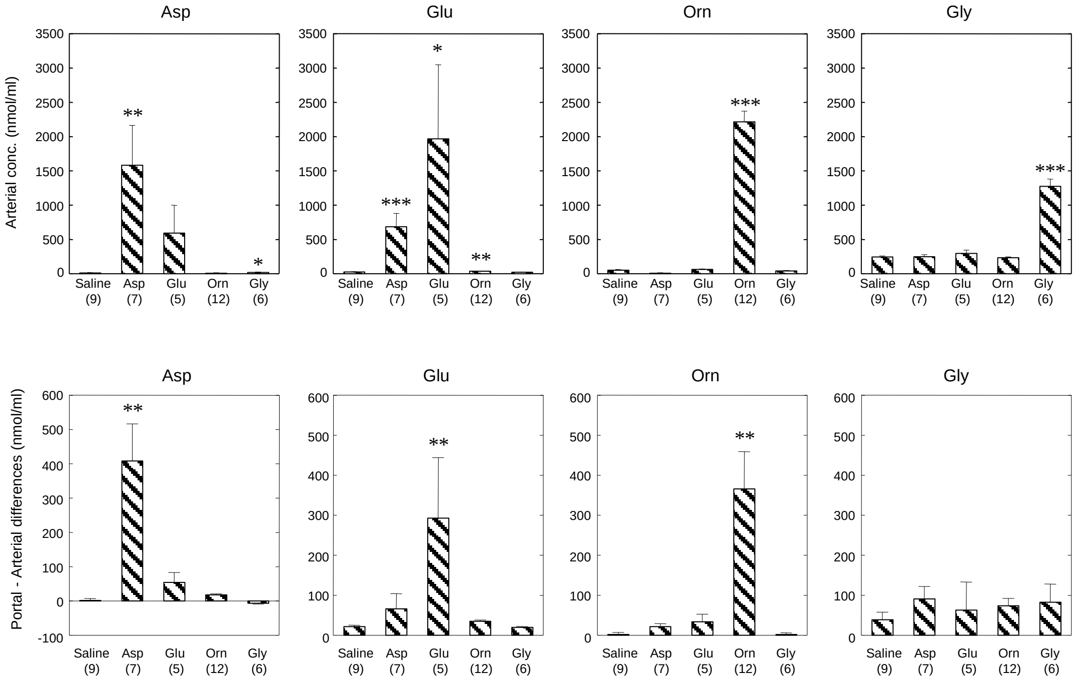

Supplementary Figure S2. Arterial concentrations (upper figures) and porta – arterial differences (lower figures) of amino acids indicated above the figures following enteral administration of amino acids listed below the figures.

Experimental procedures are the same as described in Fig. 4(a). Data are presented as mean ± SEM. Asterisks (*P<0.05, and **P<0.01) denote statistical differences from the levels when administered saline. Number of mice are shown in parenthesis.

Arterial concentrations of aspartate, glutamate, ornithine and glycine were highly increased when the corresponding amino acids were administered, and the portal – arterial differences were similarly highly increased except that administration of glycine did not increase the difference at all.

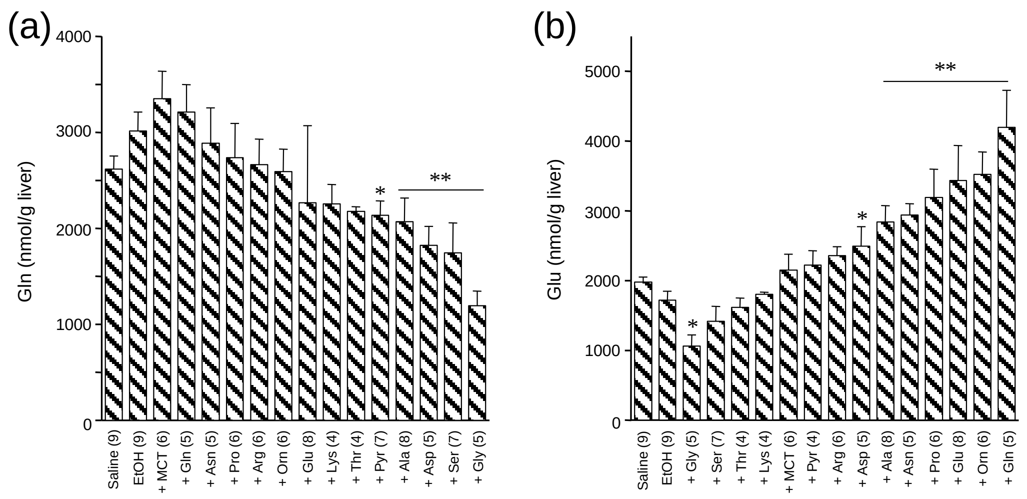

Supplementary Figure S3. Effects of amino acids on hepatic glutamine (*A*) and glutamate (*B*) in mGPD-KO mice administered 5% ethanol.

Experimental procedures are the same as described in Fig. 1. Data are presented as mean ± SEM. Asterisks (*P<0.05, **P<0.01, and ***P<0.001) denote statistical differences from the levels when administered ethanol.

Hepatic glutamine shown in Supplementary Fig. S3(a) was not affected by 5% ethanol, but decreased significantly by enteral administration of glycine, serine, aspartate, alanine and pyruvate.

Hepatic glutamate was not significantly decreased by enteral administration of 5% ethanol in mGPD-KO mice (Supplementary Fig. 3(b)). It was significantly increased by administration of glutamine, ornithine, glutamate. proline, asparagine, alanine and aspartate, while it was decreased by administration of glycine.
